# Book Reviews

**Published:** 1898-03

**Authors:** 


A Text-book of the Diseases of Women. By Henry J. Garrigues, A. M., M. D., Professor of Gynecology in the New York School of Clinical Medicine ; Gynecologist to St. Mark's Hospital in New York City, etc. Octavo, pp. 728. Containing 335 engravings and colored plates. Second edition, thoroughly revised. Philadelphia : W. B. Saunders, 925 Walnut street. 1897.

When the first edition of this excellent treatise appeared we ventured an opinion in the columns of the Journal, (Volume XXXIII., page 755, July, 1894,) the sum of which may be considered as a favorable critique of the book. We wish to reiterate our then expressed judgment, which applies with even more forcefulness to the edition before us.

Garrigues is a teacher of experience and a disciplined practitioner of excellent judgment. He has written this book with the end in view of reaching a very large audience, composed (*a*) of those physicians without previous hospital training who learn gynecology in a single post-graduate course; (*b*) those who desire to attend such an institution but think they cannot leave their patients to do so ; (*c*) general practitioners of medicine in country places who frequently acquire fame as gynecologists ; and (*d*) undergraduates. As these four classes make up a considerable proportion of the profession of medicine, existent and prospective, it follows that this work must necessarily reach a large audience if all anticipations are realised. The fact that a new edition is so promptly called for is a flattering indication of the reception given the author's first effort.

The work is now divided into three parts: first, a general division in which anatomy, physiology, etiology, examination, treatment and the like are discussed ; and second, a special division in which diseases of the vulva, of the perineum, of the vagina, of the uterus, of the tubes, of the ovaries and of the pelvis are dealt with ; and third, an appendix which takes up sterility, lack of orgasm and intestinal surgery. Garrigues is especially strong in reference to the development of the female genitals, their anatomy and physiology, and is clear, concise and instructive with reference to examination in general. One of his truisms on the latter subject in particularly striking. In speaking of Sims's position (p. 137) he says : "It is a position on the left side, but by no means is every left-side position Sims's position."

In this edition the author has paid more attention to aseptic surgery than in the former edition and in doing so has kept pace with advancements in that respect. He has modified some parts of the text and changed a number of the illustrations, eliminating those of minor importance or that have become antiquated. The patterns of instruments, too, have been revised to a certain extent and new material has been incorporated in several places. Notably, the whole surgical treatment of uterine fibroid and cancer has been re-written and simplified. The author has not hesitated in many instances to express his own opinion on the comparative value of different methods of treatment; and he has added in an appendix a short description of some of the methods employed in intestinal surgery.

It would be claiming too much to assert that this treatise is absolutely perfect, but that it presents special claims to professional favor is true. Moreover, it is a work that no gynecologist can afford to decline and it is one of the best for the general practitioner.

TWENTIETH CENTURY PRACTICE. An International Encyclopedia of Modern Medical Science. By leading authors of Europe and America. Edited by THOMAS L STEDMAN, M. D., New York City. In twenty volumes. Volume XIII., Infectious Diseases. New York: William Wood & Co. 1898.

These volumes appear with a regularity and promptitude that is almost surprising when we reflect upon the difficulties that lie in the way of publishing an encyclopedic work like this. The authors are scattered over two continents, and delays in transmission of manuscript, revision of proof and other matters of detail are almost inevitable. Hence, it is commendable to observe such prompt issuance of books as in this instance.

The most interesting section in the present volume is, in our view, the first, written by Victor C. Vaughan, of Ann Arbor. It deals with ptomains, toxins and leucomains, a subject that no one is more familiar with than this author. He first takes up bacterial poisons, then ptomains, and finally reaches food poisoning, which is a subject of paramount importance to every practising physician. Of especial moment, in this relation, are milk and cheese poisoning. These two food products are of such common use that they are found in almost every household. Frequent harm arises from their misuse when containing poisonous elements, and this volume affords an opportunity for physicians to familiarise themselves with the essential facts relating thereto.

The other sections are: Infection and immunity, by Harold C. Ernst, of Boston ; Water-borne diseases, by Ernest Hart and Solomon C. Smith, of London ; The duration of the periods of incubation and infectiousness in acute specific diseases, by Dawson Williams, of London ; Smallpox, by John William Moore, of Dublin ; Vaccinna, by P. Brouarder, of Paris ; Mumps, by Jules Comby, of Paris. An appropriate index closes this interesting and valuable volume.

HAND-BOOK OF MATERIA MEDICA, PHARMACY AND THERAPEUTICS, including the physiological action of drugs, the special therapeutics of disease, official and practical pharmacy and minute directions for prescription writing. By SAMUEL O. L. POTTER, A. M., M. D., M. R. C. P., London, Professor of the Principles and Practice of Medicine and Clinical Medicine in the College of Physicians and Surgeons of San Francisco; Medical Superintendent of St. Mark's Hospital, etc. Sixth edition. Fully revised and greatly enlarged. Royal octavo, pp. xv.—900. Philadelphia: P. Blakiston, Son & Co., 1012 Walnut street. 1897.

This treatise has long been a favorite with the medical profession, having already passed through five editions, the first of which appeared in 1886, a flattering tribute to the popularity of the author's work.

This edition is larger by one-eighth than the fifth, and so much of it has been re-written that it is almost a new book. The author asserts that forty-six new articles have been added and 170 have been re-written, while many hundred new sentences have been incorporated into the original text.

In this edition serum-therapy has been dealt with under the titles toxins, antitoxins and animal extracts. Another important subject that has received elaboration in this edition is the treatment of poisoning. Every physician should be well informed on this subject and should familiarise himself with its latest literature.

This treatise has enjoyed the confidence of many colleges that have adopted it as a text-book, and the author has industriously endeavored to make it keep pace with the progress that is making in this branch of medical science.

---

THERAPEUTICS, ITS PRINCIPLES AND PRACTICE. By H. C. WOOD, M.D., LL. D., Professor of Materia Medica and Therapeutics, and Clinical Professor of Diseases of the Nervous System in the University of Pennsylvania. A work on medical agencies, drugs and poisons, with especial reference to the relations between physiology and clinical medicine. Tenth edition, thoroughly revised. Octavo, pp. xxxi.—1033. Philadelphia: J. B. Lippincott Co. 1897.

This treatise has become so familiar to the medical world through the nine editions previously issued that little remains for the journalist to do, except to invite the attention of physicians and students to the fact that already a tenth edition is at hand. Wood's therapeutics has already been adopted as a text-book by most reputable American medical colleges; teachers and practitioners of medicine are familiar with its excellence and it is an established favorite with students. Justly so, too, for Wood was one of the first authors to treat of drugs in their relation to physiology; in other words, to establish a direct relationship between clinical and physiological medicine.

In this edition the author has made some changes in the text by placing new material in various articles throughout the work. A

few drugs of minor importance have been omitted, others new in character have been added, while the various animal drugs have been dealt with in considerable detail. Toxins and antitoxins also come in for their share. Dr. Wood is an entertaining writer, an accomplished and forceful teacher, and this present edition of his well-tried and estimable treatise brings it abreast of the therapeutics of the present.

---

TRANSACTIONS OF THE COLORADO STATE MEDICAL SOCIETY. Twenty-seventh Annual Convention. By-laws and list of members. Denver, June, 1897.

One of the most interesting features of the meeting of this society, as recorded in its proceedings, was its action in regard to the meeting of the American Medical Association to be held at Denver, June 7, 1898. The enthusiasm of the Denver physicians seemingly knew no bounds. They first proceeded to raise by subscription $2,000, pledged to the Rush monument fund by Dr. Graham, at Philadelphia, last year. This was promptly done, Dr. Graham himself subscribing $200 and several others $100 each. The next problem in finance was to raise a sufficient sum by subscription to defray the expenses of the American Medical Association, the two items being no small tax upon the physicians of Colorado.

The Colorado State Medical Society is an active, progressive and scientific body of physicians, all of which is indicated by the character of work done at its annual meeting and properly recorded in its yearly volume of transactions. The present one is no exception to the rule.

LIPPINCOTT'S POCKET MEDICAL DIRECTORY, including the pronunciation and definition of 20,000 of the principal terms used in medicine and the allied sciences, together with many elaborate tables. Edited by RYLAND W. GREENE, A. B., Editor of Lippincott's Medical Dictionary. Philadelphia and London: J. B. Lippincott & Co. 1897.

The claims of this pocket medical lexicon to professional favor are based, according to the claims of the editor made in the preface, upon its fulness, accuracy and convenience; and again, that it is up to modern requirements. Most, if not all, of these claims are made good in the text. The vocabulary is extensive in range, the definitions are for the most part clear, though concise, and the pronunciation measurably accurate; but we think it quite superfluous to adhere to the old-fashioned and obsolete methods of spelling involved in the general use of diphthongs. The book is well printed on strong thin paper, finished in gilt and handsomely and strongly bound in flexible covers. It is one of the best books of its kind for students that we have seen.

